# The low genetic diversity of the Jingmen tick virus in Guinea sheds light on the recent introduction of the virus to West Africa

**DOI:** 10.1186/s13071-025-07089-z

**Published:** 2025-11-04

**Authors:** Marat T. Makenov, Sanaba Boumbaly, Tatiana A. Bondarenko, Daria D. Skripnichenko, Namoudou Conde, Noumany Sacko, Faya Raphael Tolno, Mory Sangare, Ivan S. Kholodilov, Dmitriy V. Dubrovskiy, Mamadou Gando Diallo, Oxana A. Belova, Bonaventure Kolie, Kseniia A. Sycheva, Anna S. Kalyanova, Leno Tamba N’Fatoma, Alimou Camara, Vasily G. Akimkin, Evgeny S. Morozkin

**Affiliations:** 1 Central Research Institute of Epidemiology, Moscow, Russia; 2 Laboratory of Viral Hemorrhagic Fevers, Conakry, Guinea; 3 International Center for Research of Tropical Infections in Guinea, N’Zerekore, Guinea; 4 Chumakov Federal Scientific Center for Research and Development of Immune-and-Biological Products of RAS (Institute of Poliomyelitis), Moscow, Russia; 5 University of Kindia, Kindia, Guinea; 6 Hospital of Guéckédou, Guéckédou, Guinea

**Keywords:** Arboviruses, Genetic variation, Haplotype, Reassortant viruses, Ticks, Ixodidae, *Amblyomma*, *Rhipicephalus*, Tick-borne diseases, Livestock

## Abstract

**Background:**

Jingmen tick virus (JMTV), a segmented orthoflavi-like virus, has been recognized in recent years as a potential human and animal pathogen, with confirmed detections across Asia, Europe, Africa and South America. Recent JMTV surveillance data from Guinea has enabled detailed population genetic analysis to better understand the epidemiology of the virus in West Africa. Here, we report the results of a JMTV genetic diversity study and address specific issues of virus circulation in ticks parasitizing domestic animals.

**Methods:**

A total of 928 ticks were collected from 114 host animals across 14 Guinean prefectures, and virus isolation was subsequently attempted in the tick *Hyalomma anatolicum* (HAE/CTVM8 cell line) cell culture. All collected ticks were morphologically identified, and PCR screening for JMTV was performed. Positive samples were subjected to either complete genome sequencing or targeted fragment sequencing.

**Results:**

Our sample included nine tick species, among which only three were involved in JMTV circulation: *Rhipicephalus microplus, Rhipicephalus geigyi* and *Amblyomma variegatum*. A total of 90 tick samples tested positive for JMTV. One JMTV strain was successfully isolated in the HAE/CTVM8 cell culture. Complete genomes (all 4 segments) were sequenced for six isolates to subsequently perform genetic diversity analysis, while a fragment of segment 3 was sequenced for 64 samples to assess genetic diversity and conduct haplotype analysis. We identified and mapped three natural foci and demonstrated potential JMTV circulation in some of these over 3 years. An analysis of the ticks collected from JMTV-exposed hosts showed 40.4% PCR positivity among attached ticks. Phylogenetic and haplotype analyses demonstrated a remarkably low genetic diversity among Guinean isolates, forming a monophyletic cluster with a star-like haplotype network topology; these are patterns consistent with recent viral introduction. We also detected a reassortment event involving segment 3.

**Conclusions:**

These findings suggest that JMTV has recently been introduced to Guinea through livestock trade networks, with *R.* *microplus* ticks likely facilitating its spread. The evidence of reassortment highlights the adaptive potential of this virus in new ecosystems.

**Graphical abstract:**

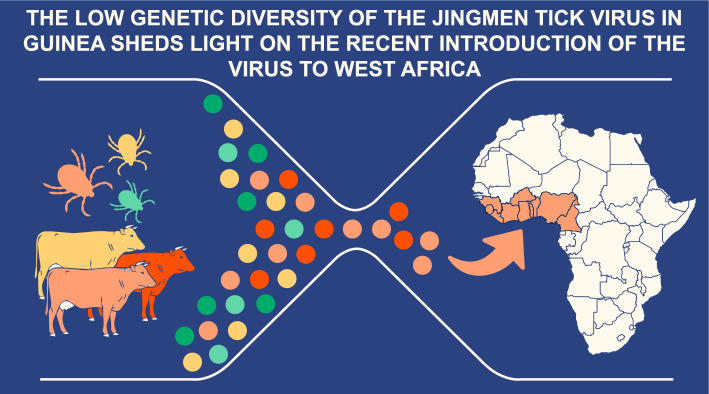

**Supplementary Information:**

The online version contains supplementary material available at 10.1186/s13071-025-07089-z.

## Background

Jingmen tick virus (JMTV) is a segmented orthoflavi-like virus representing a diverse group of viruses whose taxonomic status remains unresolved [1, 2]. This virus was first identified in 2014 in *Rhipicephalus microplus* ticks from China [3] but almost simultaneously detected in Brazil [4, 5], suggesting a potentially broad geographical distribution. Subsequent studies reported JMTV in diverse regions across the globe, including Turkey, Kosovo, Japan, Laos, the French Antilles, Uganda, Kenya and Cameroon [6–11]. In Guinea, the virus was first detected in ixodid ticks collected from domestic animals in the Kindia region in 2017 [12] and named the Kindia tick virus by the authors. A phylogenetic analysis of complete genome sequences of Kindia tick virus revealed that this virus belongs to the JMTV group [13].

Although JMTV is phylogenetically related to orthoflaviviruses (*Flaviviridae*) based on two conserved genes encoding an RNA-dependent RNA polymerase (NS5-like protein, segment 1) and a helicase (NS3-like protein, segment 3), its genome is segmented into four distinct RNA molecules [3]. This structural divergence from classical orthoflaviviruses (which possess a single positive-sense RNA genome) positions JMTV as an evolutionary bridge between segmented and non-segmented RNA viruses, offering insights into viral genome plasticity.

Following the discovery of JMTV, related viruses were identified worldwide, including Alongshan virus (ALSV) [14], Yanggou tick virus (YGTV) [15] and Takachi virus (TAKV) [9]; these viruses are collectively termed the Jingmenvirus group. This growing list of related viruses highlights their zoonotic potential, with ALSV linked to human febrile illness in China [14]. JMTV per se was detected in human blood and skin biopsies from patients with fever [8, 16], although its clinical impact requires further research.

JMTV exhibits broad vector specificity, having been detected in 26 tick species from six genera [17], with *R. microplus* implicated as a primary vector [18]. Its frequent detection in cattle raises concerns about its impact on livestock health, although its pathogenicity in animals remains uncharacterized. Importantly, the association of the virus with globally invasive tick species raises concerns over silent spread via livestock trade.

In the present study, we report the first successful isolation of Guinean JMTV strains in tick cell culture, as well as genomic characterization that identifies the low diversity of the viral population with sporadic reassortment, mapped to three endemic hotspots.

## Methods

### Tick collection and sample preparation

Ticks were collected from animals and vegetation in Guinea (West Africa) during three periods: June–July 2021, June 2022 and May–July 2024. A total of 114 animals were examined: 91 cows, five sheep, four goats, nine dogs, two striped ground squirrels (*Euxerox erythropus*), one greater cane rat (*Thryonomys swinderianus*), one red-flanked duiker (*Cephalophus rufilatus*) and one African civet (*Civettictis civetta*). Dogs, sheep and goats were examined in households, with the permission of the owner. Wild animals were examined during a chance encounter with hunters who were walking with a freshly killed animal. For cattle, ticks were collected from cows at slaughterhouses prior to slaughter. Ticks from each animal were placed in individual tubes. Ticks from vegetation were collected in the Haute Niger Reserve in June 2024 by visually detecting questing ticks on a forest path. In addition, one tick was accidentally found on grass on a trail on the northern slope of the Nimba Massif in August 2023.

The sampling approach was not standardized across prefectures due to logistical constraints inherent to Guinea's infrastructure. Remote areas presented significant challenges for proper sample storage and cold-chain transportation. Additionally, slaughterhouse capacity varied substantially between regions, with facilities in some prefectures processing two to five animals per week while those in other prefectures processed ≥ 20 animals per day. These logistical factors influenced both the geographic distribution of collections and sample sizes. Given these constraints, our sampling strategy prioritized maximizing tick species diversity across the broadest possible geographic range rather than achieving standardized collection protocols.

During fieldwork (up to 6 weeks), the ticks were stored at − 18 °C. Once in the laboratory, the tubes with ticks were stored at − 70 °C. The sampled ticks were identified to life-cycle stage and species according to their morphological characteristics [19]. Ticks were studied either individually or pooled in groups of three ticks. Subsequent prevalence analyses were conducted separately for individual and pooled samples. The pools were formed by species, sex and animal host. For subsequent analyses, ticks were washed with 70% alcohol and then rinsed twice with 0.15 M NaCl solution. Each pool was homogenized with two stainless steel beads using a TissueLyser LT homogenizer (Qiagen, Hilden, Germany) in 500 μl of 0.15 M NaCl solution, followed by DNA/RNA extraction from 100 μl of the tick suspension using the AmpliSens RIBO-prep kit (Central Research Institute of Epidemiology, Moscow, Russia) in accordance with the manufacturer’s instructions. Appropriate negative extraction controls were included. RNA was reverse-transcribed using a REVERTA-L RT kit (AmpliSens, Moscow, Russia) according to the manufacturer’s instructions.

### PCR and Sanger sequencing

For PCR screening, we used a nested PCR assay that targeted a fragment of the JMTV polymerase gene [20]. Next, for six specimens, we obtained whole-genome sequences using a panel of primers covering all four segments of the virus (see Additional file 1: Table S1, also for the amplification program for each primer pair. To study genetic diversity, we amplified a fragment of segment 3 using primers Kind-NS3-F-1210 and Kind-NS3-R-2070 (Additional file 1: Table S1). To detect JMTV in tick cell culture, we amplified a fragment of segment 2 using primers JMTV_test_F1 and JMTV_test_R1 (Additional file 1: Table S1). Tick species identification was confirmed by sequencing the cytochrome* c* oxidase I (COI) gene fragment using previously published primers [21].

The purified PCR products were sequenced bidirectionally using a BigDye Terminator v1.1 Cycle Sequencing kit (Thermo Fisher Scientific, Waltham, MA, USA) on an Applied Biosystems 3500xL Genetic Analyzer (Applied Biosystems, Thermo Fisher Scientific, Foster City, CA, USA). The sequences obtained were deposited in NCBI GenBank under the following accession numbers: PP779169–PP779192 for JMTV, and PV461569–PV461589 for ticks.

### Virus isolation

To isolate the virus, we used cell lines derived from embryos of the tick *Hyalomma anatolicum* (HAE/CTVM8) provided by the Tick Cell Biobank (Liverpool, UK). The tick cell line was maintained at 28 °C in L-15 (Leibovitz) medium (Chumakov FSC R&D IBP RAS, Moscow, Russia) supplemented with 10% tryptose phosphate broth (Difco, Detroit, MI, USA), 20% fetal bovine serum (Gibco, Invitrogen, Thermo Fisher Scientific, Carlsbad, CA, USA), 2 mM L-glutamine and antibiotics, as described in a previous study [19]. Prior to infection, the HAE/CTVM8 cells were seeded in 96-well plates (TPP Techno Plastic Products AG, Trasadingen, Switzerland) in 200 µl of complete medium and incubated at 28 °C. One week later, cells were infected by adding 20 μl of unfiltered tick homogenate and incubated at 28 °C. Medium was changed at weekly intervals by the removal and replacement of 100 μl; the spent medium was used to harvest the virus. The culture medium was checked each week by reverse transcription PCR (RT-PCR) to detect the virus using primers JMTV_test_F1 and JMTV_test_R1 (Additional file 1: Table S1).

### Data analysis

For the phylogenetic analysis, we used JMTV sequences available in GenBank as of 1 February 2024 that met the following two criteria: (i) tick-derived isolates with (ii) complete whole-genome sequences of all segments. Multiple sequences were aligned with the ClustalW algorithm using default settings in the Mega X package [22]. The sequences were then trimmed to exclude poor quality bases and obtain uniform sizes. The Mega X package was used for maximum likelihood phylogenetic analysis. Trees were constructed for each segment and a fragment of segment 3, and later visualized by ITOL [23]. Neighbor-joining trees were constructed in MEGA X to assess potential reassortment.

Multiple sequences extracted from the alignments were used to construct haplotypes using the DnaSP software v5.10.01 [24]. Genetic variation indices, including the number of haplotypes (*H*), the number of segregating sites (*S*), haplotype diversity (*Hd*) and nucleotide diversity (*π*), were calculated for each JMTV segment and for a fragment of segment 3 using DnaSP. The haplotype network was constructed using the minimum spanning method in the PopART (Population Analysis with Reticulate Trees) software [25]. In this study, we define viral haplotypes as distinct genetic variants of the RNA genome characterized by a specific combination of nucleotide polymorphisms.

We employed the following strategy to identify reassortment events:

1. Alignments were created with identical numbers of sequences and list of isolates for each of the four segments.
2. For each segment, we determined the number of haplotypes, the number of haplotypes represented by only one sequence in the alignment and those represented by multiple identical sequences.
3. In the absence of reassortment events, we expected equal numbers of haplotypes for each segment, as well as identical clustering patterns of identical haplotypes.
4. When haplotype numbers did not match, we suspected reassortment events and proceeded with phylogenetic analysis for manual identification of reassortment evidence. Using MEGA X, we constructed neighbor-joining trees without evolutionary models (the evolutionary distances were computed using the p-distance method) and calculated p-distances.
5. To assess sufficient numbers of substitutions, we used Fisher's exact test to evaluate the hypothesis that the observed number of substitutions significantly differed from zero.

The confidence intervals for prevalence estimates were calculated in R using the PropCIs package [26]. We assessed the minimum infection rate (MIR) as the ratio between the number of positive pools and the total number of specimens tested [27].

## Results

### Identification of the JMTV endemic transmission hotspots in Guinea

**Fig. 1:**
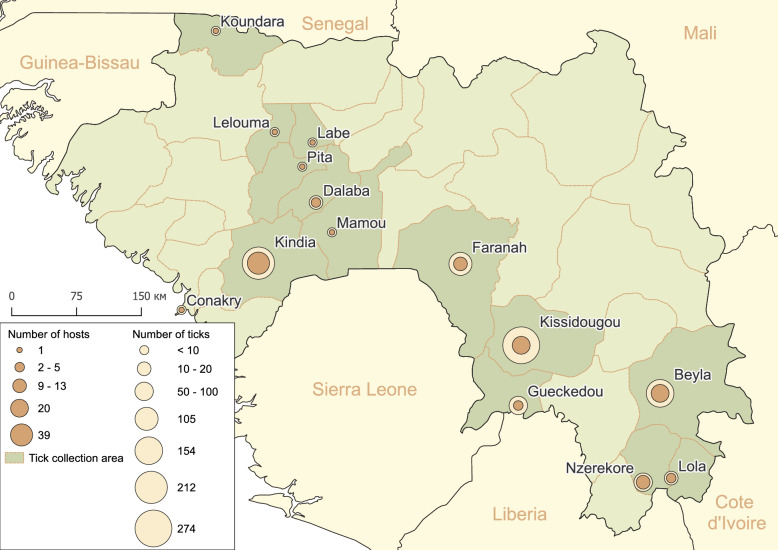
The geographic distribution of tick sampling sites, with total tick counts and livestock examined per location

A total of 928 ticks, including 856 ticks from 114 host animals and 72 ticks from vegetation, were collected and grouped into 689 pools (Table 1). JMTV was subsequently in 90 tick pools collected from 20 animals in three prefectures (Kissidougou, Beyla and Kindia prefectures). All of the JMTV-positive ticks were collected from cows only. In Kissidougou Prefecture, all of the examined cows (*n* = 20) were from the village of Yende-Millimou (8.88957 N, 10.16935 W), and 40% of these had JMTV-positive ticks; JMTV-positive ticks had also been collected from cattle in this village in 2022 and 2024. This result indicates that a natural focus of the virus has been formed in the vicinity of Yende-Millimou (Fig. 1). A similar result was obtained in Beyla Prefecture, where ticks were collected from cows in four villages, with JMTV-positive ticks detected in three of these (Table 1). The distance between these villages ranges from 36 to 73 km, and our findings demonstrate that JMTV is circulating across these extensive areas. In the Kindia region, we examined 39 domestic animals and collected 212 ticks. Only one pool, containing three male *Amblyomma variegatum* ticks, tested positive for JMTV (MIR in the region was 0.5) (Table 1). Our data are insufficient to confirm the presence of a natural hotspot in Kindia, but having added available data from GenBank and other publications, we suggest that Kindia may represent an endemic hotspot for JMTV circulation (see section Haplotype analysis).

**Table 1:** The number of studied ticks and the results of the PCR screening of Jingmen tick virus

Prefecture/host animal	Number of investigated host animals	Number of collected ticks	Number of tick pools formed	Number of JMTV-positive tick pools	Number of JMTV-exposed host animals^a^
Conakry					
Cattle	1	5	3	0	0
Koundara					
Cattle	1	7	5	0	0
Kindia					
Cattle	31	182	116	1	1 (3.2%)
Goat	3	9	7	0	0
Dog	5	21	17	0	0
Labe					
Cattle	1	4	4	0	0
Lelouma					
Cattle	1	5	3	0	0
Mamou					
Cattle	1	2	2	0	0
Pita					
Cattle	1	1	1	0	0
Dalaba					
Cattle	1	12	4	0	0
Goat	1	6	4	0	0
Faranah					
Cattle	9	30	28	1	1 (11.1%)
Dog	2	2	2	0	0
*Cephalophus rufilatus*	1	2	2	0	0
Vegetation	NA	71	27	0	0
Kissidougou					
Cattle	20	274	213	50	8 (40.0%)
Gueckedou					
Cattle	2	17	13	0	0
*Civettictis civetta*	1	46	46	0	0
* Thryonomys swinderianus*	1	6	6	0	0
Beyla					
Cattle	18	129	115	38	10 (55.6%)
Sheep	2	25	23	0	0
Nzerekore					
Cattle	2	14	8	0	0
Sheep	3	18	10	0	0
Dog	2	13	5	0	0
*Euxerus erythropus*	2	10	8	0	0
Lola					
Cattle	2	16	16	0	0
Vegetation	NA	1	1	0	0
Total	114	928	689	90	20

^a^In this context, JMTV-exposed host animals refer to animals from which JMTV-positive ticks were collected. The host animals themselves were not tested in this study

*JMTV* Jingmen tick virus, *NA* not applicable

### Virus-vector association

JMTV was detected in ticks of three species: *A.* *variegatum*, *R.* *microplus*, and *Rhipicephalus geigyi* (Table 2). One JMTV-positive tick was damaged during extraction from the host cow, preventing morphological identification to the species level. Sequencing of the COI gene confirmed the tick's classification within the genus *Rhipicephalus* (excluding the *Boophilus* subgenus). However, the absence of matching reference sequences in GenBank precluded precise species determination. The remaining tick species were JMTV-negative. *Rhipicephalus microplus* ticks were significantly more likely to test positive for JMTV than *A.* *variegatum* and *R.* *geigyi* ticks (chi-square [*χ*^2^] = 38.47,* df* = 2, *p*-value < 0.001).

**Table 2:** Prevalence of Jingmen tick virus by tick species

Tick species	Number of ticks collected	Number of pools formed	Number of hosts	PCR-positive ticks on JMTV, number; % (95% CI)
Unpooled samples				
*Amblyomma variegatum*	361	361	87	34; 9.4% (6.6–12.9%)
*Amblyomma paulopunctatum*	1	1	NA^a^	0
*Amblyomma splendidum*	1	1	NA	0
*Rhipicephalus microplus*	130	130	34	42; 32.3% (24.4–41.1%)
*Rhipicephalus geigyi*	35	35	20	5; 14.3% (4.8–30.3%)
*Rhipicephalus (Boophilus)* sp.	11	11	6	0
*Rhipicephalus sanguineus*	7	7	3	0
*Rhipicephalus senegalensis*	4	4	NA	0
*Rhipicephalus *sp.	6	6	3	1
*Hyalomma truncatum*	3	3	3	0
*Haemaphysalis leachi*	9	9	4	0
Pooled samples				
*A. variegatum*	261	87	48	8; 3.1%^b^
*R. microplus*	3	1	1	0
*R. geigyi*	6	2	1	0
*Rhipicephalus (Boophilus) *sp.	3	1	1	0
*R. sanguineus*	18	6	4	0
*R. senegalensis*	66	22	NA	0
*Haem. leachi*	3	1	1	0

* CI* Confidence interval, *JMTV* Jingmen tick virus

^a^*NA* Not applicable. These ticks were collected from vegetation

^b^The prevalence for pooled data was calculated as the minimum infection rate (MIR); see section Data analysis

### Ticks co-feeding and JMTV

Next, we focused exclusively on the 20 animals from which JMTV-positive ticks were collected. The infection status of these animals remains unknown because no blood or skin samples were collected. A total of 223 adult ticks of three species at varying stages of engorgement were collected from these animals. Viral RNA was detected in 40.4% of these ticks; a sufficient amount of virus was not acquired from the remaining 59.6% of ticks during co-feeding for viral RNA to be detected by nested PCR. The proportion of positive ticks varied depending on the tick species (Table 3): 46.7% of co-fed *R.* *microplus* were PCR-positive compared to 34.1% of *A.* *variegatum*; this difference was marginally significant (*χ*^2^ = 3.41,* df* = 1, *p*-value = 0.06). Due to insufficient sample size, we were unable to estimate the proportion of positive ticks for *R.* *geigy*.

**Table 3:** Proportion of infected ticks out of the total number of ticks collected from Jingmen tick virus-exposed cows (*n* = 20)

Tick species	Total number of ticks collected	JMTV-positive ticks		
		Number	Percentage	95% CI
*Amblyomma variegatum*	123	42	34.1	25.8–43.2
*Rhipicephalus microplus*	90	42	46.7	36.1–57.5
*Rhipicephalus geigyi*	6	5	83.3	11.8–88.2
*Boophilus* sp.	3	0	0.0	Not calculated
*Rhipicephalus * sp.	1	1	100.0	Not calculated
Total	223	90	40.4	33.9–47.1

In this context, JMTV-exposed host animals refer to animals from which JMTV-positive ticks were collected. The host animals themselves were not tested in this study

* CI* Confidence interval, *JMTV* Jingmen tick virus

### Virus isolation

We successfully isolated a JMTV strain named Yende-Millimou-125 in the HAE/CTVM8 cell line culture. Viral persistence was monitored weekly by collecting aliquots of culture supernatant and detecting viral RNA via PCR with primers JMTV_test_F1 and JMTV_test_R1.

The virus was maintained for 14 weeks (100 days), corresponding to a cumulative 16384-fold dilution of the original infectious homogenate. Despite this extreme dilution, RNA extraction followed by RT-PCR consistently yielded target amplicons, with JMTV identity confirmed by Sanger sequencing. Notably, no cytopathic effects were observed throughout the 100-day culture period.

### Phylogenetic analysis of JMTV

**Fig. 2:**
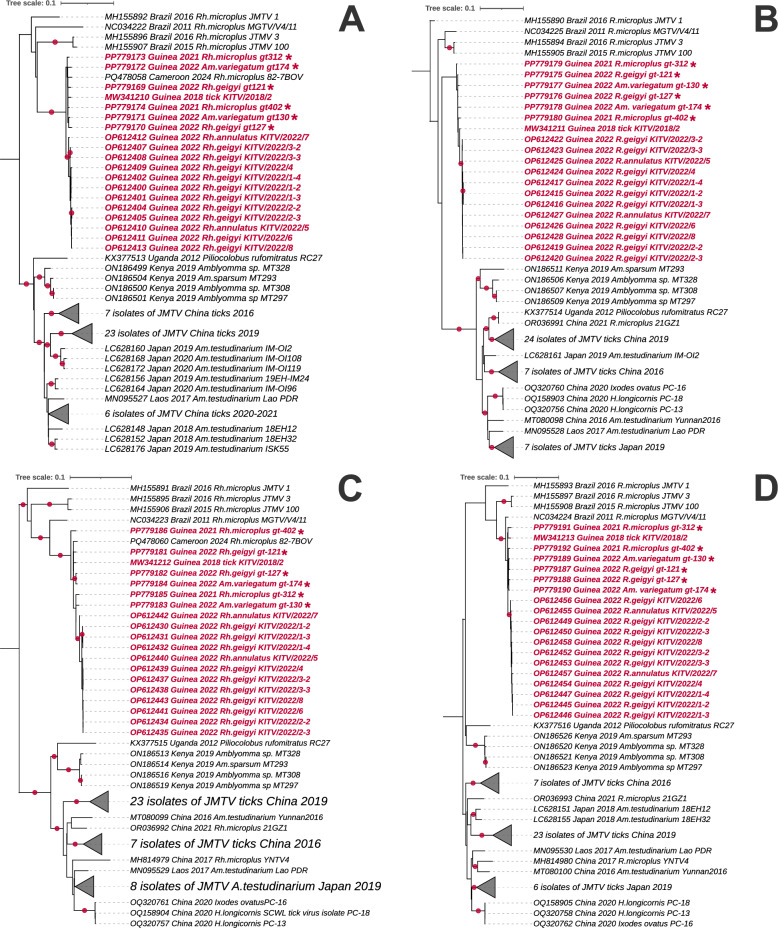
The maximum likelihood trees of JMTV segments. Trees were drawn to scale, with branch lengths measured in the number of substitutions per site. Bootstrap support values > 90% are indicated by solid circles at branch nodes. Alongshan virus strain Miass519 was used as an outgroup (not shown in the figure). Each tree represents one segment. **A** Segment 1: 2694 nt, 75 sequences; model: General Time Reversible + Gamma + Invariant Sites. **B** Segment 2: 2535 nt, 74 sequences; model: Tamura-Nei + Gamma + Invariant Sites. **C** Segment 3: 1972 nt, 75 sequences; model: General Time Reversible + Gamma + Invariant Sites. **D** Segment 4, 2469 nt, 74 sequences; model: Tamura-Nei + Gamma + Invariant Sites. JMTV, Jingmen tick virus; nt, nucleotide

Phylogenetic analysis of nearly complete sequences of all four segments showed that the JMTV isolates were closest to isolates named Kindia tick virus obtained previously from Guinean ticks (Fig. 2). All of the Guinean JMTV isolates shared the highest similarity with strains from Brazil across all four segments and formed a separate cluster. The isolate from Cameroon also belongs to this group (Fig. 2a, c). East African isolates (from Uganda and Kenya) formed a separate cluster that was more closely related to Asian strains than to those from West Africa.

A comparative analysis of within-group genetic distances demonstrated significantly constrained diversity in Guinean JMTV isolates across all the genomic segments. The average p-distance values for segments 1–4 was 0.0087, 0.0077, 0.0099 and 0.0102 in Guinean isolates versus 0.0384, 0.0393, 0.0399 and 0.0401 in Chinese isolates, representing a 3.9- to 5.2-fold reduction in nucleotide diversity (Additional file 1: Table S2).

### Genetic diversity

We aligned sequences from 74 JMTV isolates—including six isolates obtained in the present study—for which complete genome sequences of all four segments were available (Additional file 2: Table S3). The total alignment length for each segment was constrained by the shortest sequence in the alignment. The analysis revealed a high level of genetic diversity in JMTV: the number of haplotypes in the set of 74 sequences ranged from 55 to 61 haplotypes across segments, with haplotype diversity exceeding 0.98 (Table 4). These diversity metrics were calculated for isolates obtained from 11 tick species and one red colobus monkey (*Piliocolobus rufomitratus*), representing eight different countries. The broad geographical distribution of samples and diverse vector species composition account for the observed high genetic variability.

**Table 4:** Genetic diversity of the Jingmen tick virus by segments

Segment	Length of alignment^a^, nt	Genetic diversity statistics/indices^b^				
		*S*	*π* (SD)	*H*	*Hd* (SD)	Tajima’s* D*
All isolates (*n* = 74)						
Segment 1	2681	849	0.0724 (0.0026)	60	0.990 (0.005)	− 0.0519
Segment 2	2413	725	0.0733 (0.0025)	61	0.994 (0.004)	0.1982
Segment 3	1953	615	0.0777 (0.0031)	59	0.992 (0.005)	0.2183
Segment 4	2451	715	0.0698 (0.0020)	55	0.987 (0.006)	0.1461
Chinese isolates (*n* = 36)						
Segment 1	2690	413	0.0377 (0.0047)	28	0.970 (0.019)	− 0.0897
Segment 2	2432	381	0.0377 (0.0052)	30	0.984 (0.012)	− 0.1388
Segment 3	1977	342	0.0385 (0.0049)	30	0.981 (0.015)	− 0.3940
Segment 4	2452	403	0.0402 (0.0057)	25	0.960 (0.020)	− 0.1120
Guinean isolates (*n* = 18)						
Segment 1	2687	93	0.0080 (0.0013)	14	0.967 (0.030)	− 0.8768
Segment 2	2444	61	0.0068 (0.0012)	14	0.967 (0.030)	− 0.2376
Segment 3	1966	75	0.0087 (0.0016)	13	0.954 (0.034)	− 0.9900
Segment 4	2453	91	0.0078 (0.0013)	14	0.967 (0.030)	− 1.1629

*nt* Nucleotide,* SD* standard deviation

^a^Length of alignment showed excluding sites with gaps/missing data

^b^*S* Number of polymorphic (segregating) sites, *π* nucleotide diversity, *H*, number of haplotypes, *Hd* haplotype diversity

Subsequently, we focused exclusively on Guinean JMTV isolates, analyzing an alignment of 18 near-complete genomes. This subset demonstrated a significantly lower genetic diversity across all the segments, with the number of haplotypes ranging from 13 to 14; nucleotide diversity (*π*) varying between 0.0068 and 0.0087; and the number of polymorphic (segregating) sites ranging from 61 to 93.

We used the dataset of Chinese JMTV for comparative purposes. China was chosen due to the substantial number of available sequences, which far exceeds the number available from other countries. Importantly, we intentionally confined the Chinese dataset to tick-derived sequences to avoid potential overestimation of genetic polymorphism that might occur with sequences obtained from vertebrate hosts. The Chinese isolates exhibited moderately high genetic diversity with the following indices: 342–413 polymorphic sites, 25–30 haplotypes and nucleotide diversity (*π*) ranging from 0.0377 to 0.0402; these values were substantially higher than those observed in the Guinean isolates. Tajima's* D* for all the segments showed no significant deviation from zero, providing no evidence for selection pressure. Similar results were obtained for Guinean and Chinese isolates—no significant deviations of Tajima's* D* from zero (Table 4).

Since segment 3 (encoding the orthoflavivirus-like NS3 protein) showed the highest nucleotide diversity both in the complete dataset and among Guinean isolates specifically, this genomic region was selected for detailed genetic diversity analysis (see section Haplotype analysis).

### Haplotype analysis

**Fig. 3:**
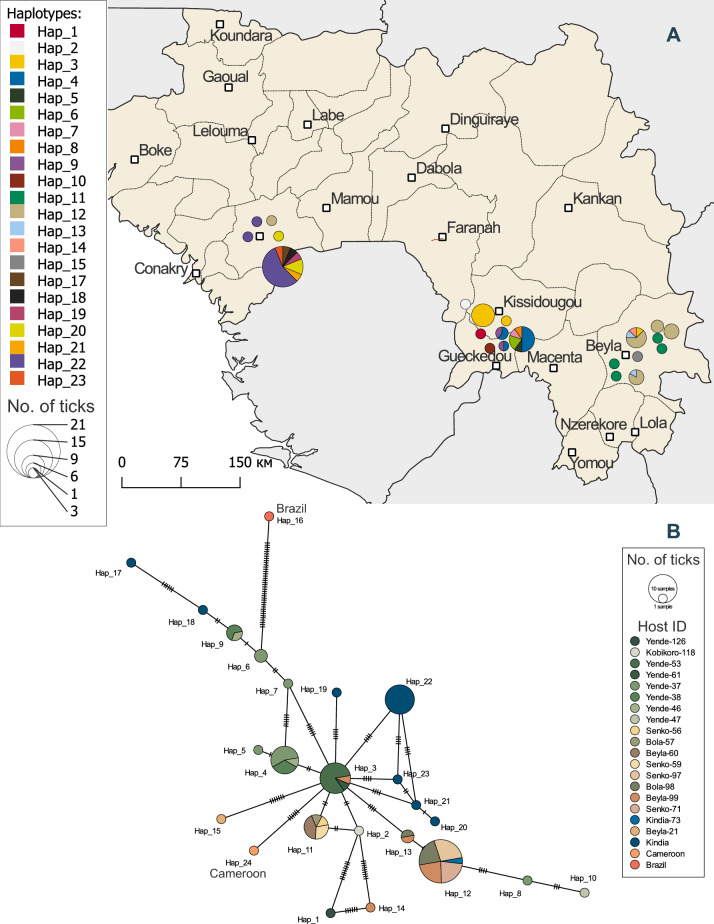
Haplotype analysis of a 716-nt fragment of segment 3 of the JMTV. **a** Map of Guinea, depicting each host animal (circles) from which JMTV-positive ticks were collected. Colors represent different haplotypes, and circle size shows the number of ticks with a given haplotype collected from each host animal. **b** Minimum spanning haplotype network. Circles represent haplotypes, and colors depict host animals. Each hatch mark on a line corresponds to 1 mutation. JMTV, Jingmen tick virus

We sequenced an approximately 800-nt fragment of segment 3 (encoding the orthoflavivirus-like NS3 protein) from 64 Guinean samples and supplemented those with 18 additional Guinean sequences of this region from GenBank. The resulting alignment comprised 82 sequences with a total length of 716 nt. Subsequent analysis of this expanded dataset revealed greater haplotype diversity compared to whole-genome analyses (Fig. 3), identifying 22 distinct haplotypes with a haplotype diversity (*Hd*) of 0.881 (standard deviation [SD] 0.021).

We mapped the geographic coordinates of JMTV-positive samples to visualize the regional distribution of viral haplotypes. In addition to the two previously described natural foci, we identified the Kindia Prefecture as a third endemic territory. Collectively, three epidemiologically distinct foci were resolved (Fig. 3a):

− the Kissidougou Prefecture, centered around Yende-Millimou village (including 1 isolate from Kobikoro village located 52 km from Yende-Millimou)
 − the Beyla Prefecture (encompassing Beyla, Sinko and Bola villages)
 − the Kindia Prefecture, where 18 of 19 sequences were obtained from GenBank with only prefecture-level geographic data available.

The most diverse focus was observed near Yende-Millimou village (10 haplotypes). Kindia Prefecture and Beyla Prefecture yielded eight and six JMTV haplotypes, respectively. Notably, Hap_3 was present in both the Beyla and Yende-Millimou villages, while Hap_12 was found in ticks from the Beyla, Bola, Sinko and Kindia prefectures. The most prevalent haplotypes were Hap_12 and Hap_11, detected in ticks collected from five and four host animals, respectively.

Network analysis revealed a star-like topology centered around Hap_3, with radiating branches connecting to haplotypes from the Kindia, Yende-Millimou and Beyla prefectures (Fig. 3b). As the graph shown in Fig. 3b was undirected, we could not establish haplotype chronology. Phylogenetic analysis of segment 3 prompted inclusion of an ancestral JMTV sequence from Brazil (isolate MGTV/V4/11, accession: JX390985) as Hap_16. This sequence connected via a single edge to Hap_6 (*R.* *microplus* and *R.* *geigyi* ticks from Yende-Millimou), which subsequently gave rise to Hap_7 through two substitutions, ultimately linking to the central Hap_3. While the star-like topology might suggest recent population expansion, Tajima's* D* for this fragment was non-significant (*D* = − 1.16443, *p*-value > 0.1).

Haplotype analysis yielded additional important findings. First, individual host animals harbored ticks with different JMTV haplotypes; for example, host Yende-38 (a cow from Yende-Millimou) carried three newly attached *A.* *variegatum* females with Hap_4 and two engorged *R.* *microplus* females with Hap_9 (Table 5). It is worth noting here that *R.* *microplus* is a one-host tick species that typically remains on their host for up to 3–4 weeks, while *A.* *variegatum* is a two-host tick species whose immature stages parasitize small mammals, while adult ticks infest large animals. Based on these findings, the collected *Amblyomma* adults had only just attached to the cattle, showing no signs of prolonged feeding, suggesting that the *A.* *variegatum* females likely acquired the virus before attaching to this host—during their nymphal or larval stages.

**Table 5:** Detection of multiple Jingmen tick virus haplotypes in ticks collected from individual cows

Host ID	Tick species	Number of ticks/pool	Sex/stage and feeding status	Haplotype
Yende-37	*Amblyomma variegatum*	1	Partially engorged female	Hap_4
	*A. variegatum*	1	Unfed female	Hap_4
	*A. variegatum*	1	Male	Hap_5
	*Rhipicephalus microplus*	1	Engorged female	Hap_4
	*R. microplus*	1	Engorged female	Hap_4
	*R. microplus*	1	Engorged female	Hap_4
	*R. microplus*	1	Engorged female	Hap_6
	*R. microplus*	1	Engorged female	Hap_7
	*Rhipicephalus geigyi*	1	Engorged female	Hap_8
	*R. geigyi*	1	Engorged nymph	Hap_6
Yende-38	*A. variegatum*	3	Partially engorged females	Hap_4
	*R. microplus*	1	Engorged female	Hap_9
	*R. microplus*	1	Engorged female	Hap_9
Yende-46	*R. microplus*	1	Engorged female	Hap_4
	*R. microplus*	1	Engorged female	Hap_9
Bola-98	*A. variegatum*	3	Male	Hap_12
	*A. variegatum*	1	Partially engorged female	Hap_12
	*A. variegatum*	1	Unfed female	Hap_12
	*A. variegatum*	1	Unfed female	Hap_12
	*A. variegatum*	1	Unfed female	Hap_13
Beyla-99	*A. variegatum*	1	Unfed female	Hap_12
	*A. variegatum*	3	Partially engorged females	Hap_12
	*R. microplus*	1	Partially engorged female	Hap_12
	*R. microplus*	1	Engorged female	Hap_12
	*R. microplus*	1	Engorged female	Hap_12
	*R. microplus*	1	Partially engorged female	Hap_3
	*R. microplus*	1	Engorged female	Hap_13
	*R. microplus*	1	Engorged female	Hap_14

Host Yende-46 (another cow from the same village) harbored two engorged *R.* *microplus* females containing distinct haplotypes (Hap_4 and Hap_9). These female ticks fed simultaneously on the same host animal but contained different viral haplotypes. The JMTV infection status of the host remained undetermined due to unavailable blood/skin biopsy samples, preventing evaluation of systemic infection or haplotype-specific dissemination. In addition, high diversity was observed in two animals (host ID: Beyla-99 and Yende-37; see Table 5), which hosted ticks containing four and five distinct JMTV haplotypes, respectively (Table 5). The detection of co-feeding ticks carrying divergent JMTV variants on the same host suggests conditions favorable for reassortment. This provides empirical support for segment exchange between co-circulating virus variants.

Furthermore, we detected at least two distinct genetic variants of JMTV co-infecting a single tick. The engorged nymph of *R.* *geigyi*, collected from a cow in Yende-Millimou, exhibited heterogeneous peaks in Sanger sequencing chromatograms (Additional file 3: Figure S1), indicating concurrent infection with at least two viral variants. These heterogeneities were observed across all four genomic segments, with seven, 11, 25 and five polymorphic sites identified in segments 1 through 4, respectively.

### Evidence for reassortment in Guinean JMTV isolates

**Fig. 4:**
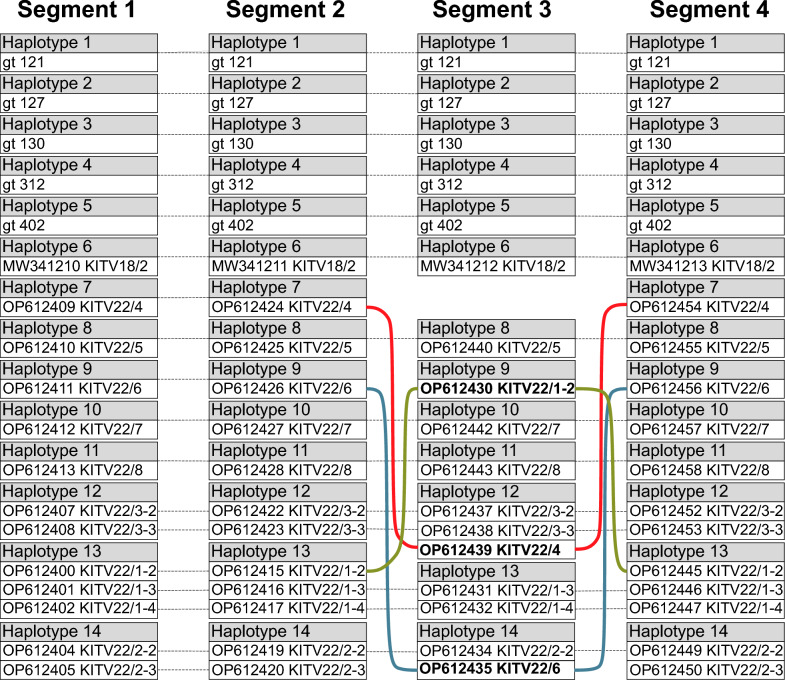
Visualization of the haplotype distribution among Guinean JMTV isolates. Blocks represent individual haplotypes; gray dashed lines indicate haplotype connections between segments where no evidence of reassortment was detected; colored solid lines highlight discordant connections indicative of either reassortment (red and blue) or point mutations (green). JMTV, Jingmen tick virus

We observed identical haplotype numbers (*h* = 14) for segments 1, 2 and 4 in Guinean samples, while segment 3 showed one fewer haplotype (*h* = 13) (Table 4). This discrepancy suggests possible reassortment. Visualization of the haplotype distribution revealed that our sequences were unique compared to other Guinean JMTV sequences (Fig. 4), preventing direct reassortment analysis for these isolates.

However, we did identify potential reassortment events in previously reported Guinean JMTV sequences. The dataset comprised 18 sequences, with 11 unique haplotypes for segment 1 (Fig. 4):

 − Haplotype 12 included two sequences: isolates KITV2022/3-2 and KITV2022/3-3
 − Haplotype 13 comprised three sequences: KITV2022/1–2, KITV2022/1–3, and KITV2022/1–4
 − Haplotype 14 contained two sequences: KITV2022/2-2 and KITV2022/2–3.

While segments 1, 2, and 4 showed identical clustering patterns, segment 3 displayed divergent haplotype assignments (Fig. 4):

 − Sequence KITV2022/6 (segment 3) grouped with haplotype 14, while KITV2022/4 (segment 3) clustered with haplotype 12 (suggesting reassortment)
 − Additionally, sequence KITV2022/1–2, previously part of haplotype 13, formed a separate haplotype in segment 3. However, distance analysis revealed only a single nucleotide substitution (*P*-distance = 0.0005) distinguishing KITV2022/1–2 from KITV2022/1–3 and KITV2022/1–4. Hence, this divergence was likely to result from mutation rather than reassortment.

**Fig. 5:**
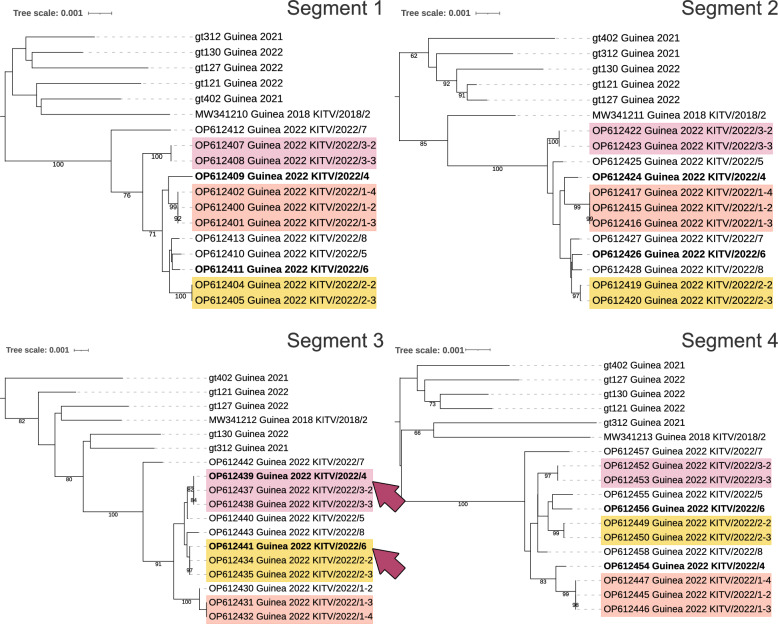
Neighbor-joining phylogenetic trees of Guinean JMTV isolates for each genomic segment. Evolutionary distances were computed using the p-distance method, expressed as the number of base substitutions per site. The analyzed alignments contained 2802 nucleotides for segment 1, 2471 nucleotides for segment 2, 1987 nucleotides for segment 3 and 2453 nucleotides for segment 4. Bootstrap support values > 90% are indicated by solid circles at branch nodes. Sequences exhibiting evidence of reassortment are highlighted in bold, with pink arrows specifically denoting reassorted isolates in segment 3. JMTV, Jingmen tick virus

To confirm reassortment through phylogenetic methods, we constructed neighbor-joining trees (Fig. 5), maintaining the same alignment lengths for each segment as those used in the haplotype analysis to preserve haplotype numbers. The analysis revealed discordant clustering of isolates KITV2022/4 and KITV2022/6 in segment 3 compared to their placement in segments 1, 2 and 4. However, distance metrics showed only minor nucleotide differences between KITV2022/6 and reference isolates KITV2022/2-2/KITV2022/2–3 across segments 1, 2 and 4 (4, 2, and 4 substitutions corresponding to p-distances of 0.15%, 0.08% and 0.16%, respectively). Fisher's exact test indicated that these genetic distances did not significantly differ from zero (*p*-values 0.12, 0.50, 0.12), leaving the reassortment status of KITV2022/6 inconclusive. In contrast, the isolate KITV2022/4 exhibited more substantial divergence from KITV2022/3-2 and KITV2022/3-3, with 10, three and six substitutions in segments 1, 2 and 4 (p-distances: 0.27%, 0.12%, 0.24%). Fisher's exact test confirmed statistically significant distances for segments 1 and 4 (*p*-values = 0.002 and 0.031). Combined with phylogenetic topology, these results provide compelling evidence for segment 3 reassortment in KITV2022/4. For the remaining Guinean sequences, reliable detection of reassortment events was not possible due to the limited number of available sequences.

## Discussion

JMTV was initially discovered in 2014 in China, and first identified in ticks from Guinea's Kindia Prefecture in 2020 [12]. Subsequent studies confirmed JMTV detection in Kindia during 2021 [13, 28], indicating the establishment of a natural virus focus in this region, with ongoing circulation among ticks and domestic animals. The findings of the investigation reported here extend these observations by identifying additional endemic areas in Guinea. In our Kindia samples, only one tick was JMTV-positive (0.5% prevalence), with this low prevalence suggesting either limited sample size or uneven viral distribution, with the formation of spatially discrete foci. Beyond Kindia, we identified two additional JMTV foci: the Yende-Millimou village area and the Beyla Prefecture. These findings demonstrate a broader geographical distribution of JMTV in Guinea than previously recognized. Future studies should map livestock farm distributions and land use patterns across the study area.

Regular livestock transport by Guinean farmers facilitates the spread of infected ticks to new areas. This activity extends beyond national borders and follows well-documented West African trade networks [29, 30]. Guinea's cattle imports originate predominantly from Mali, with some from Burkina Faso, mirroring the introduction route of the invasive tick *R.* *microplus* through Malian cattle [31]. We hypothesize similar introduction routes for JMTV, suggesting widespread West African distribution, although current surveillance reports no detections in Mali or Burkina Faso. Supporting evidence comes from recent JMTV identification in Cameroon [11]. Despite separation by 2000 km and five intervening countries, Cameroonian JMTV is genetically similar to Guinean variants, suggesting connections through long-distance tick movement via livestock transport. However, trade records reveal no direct cattle exchange between Guinea and Cameroon [30]. Cameroon sources livestock from Nigeria, which in turn imports from Niger, Burkina Faso and Benin. Hence, JMTV probably occurs in ticks throughout West Africa east of Guinea. Tick studies in other West African countries, as well as targeted studies of livestock transported during cross-border trade, are therefore needed.

JMTV was detected in three out of nine tick species sampled: *A.* *variegatum*, *R.* *geigyi* and *R.* *microplus*, plus one unidentified *Rhipicephalus* sp. (Table 2). The first two species represent the most abundant ticks parasitizing domestic animals in Guinea [13, 32, 33], whereas *R.* *microplus* is an invasive species first reported in Guinea in 2018 [31]. The absence of JMTV in other tick species may reflect their limited representation in our sample. Previous studies have identified JMTV in ticks of the *Boophilus* subgenus, including *R.* *microplus* [3, 5, 34]*, R.* *geigyi* and *R. annulatus* [13], and in *Amblyomma* genus, including *A.* *sparsum* in Kenya [10] and *A.* *testidinarium* in Japan and China [9]. A phylogenetically similar virus was detected in *A. variegatum* from the French Antilles [35]. In all cases, the virus was found in engorged ticks removed from host animals. JMTV has also been detected in bovine blood [9, 28, 34], making it impossible to determine whether ticks acquired the virus through host blood or had been previously infected before attachment. Nevertheless, the growing reports of JMTV in ixodid ticks in different countries strongly suggest their involvement in viral circulation. Our study revealed significantly higher infection rates in *R. microplus* than in *A.* *variegatum* (Table 2), potentially indicating its role as the primary vector. This hypothesis is further supported by the overlapping geographical distributions of JMTV detections. To date, JMTV has been detected in Brazil, China, Laos, Japan, Uganda, Kenya, Guinea and Cameroon—all countries where the presence of *R.* *microplus* has been confirmed [36]. Future laboratory studies should evaluate vector competence and the efficiency of transstadial/transovarial transmission among different ixodid species. Additional evidence supporting *R. microplus* as a competent vector for JMTV comes from its successful replication in BME/CTVM23 cell line cultures [16].

Several transmission routes have been described for tick-borne pathogens. In the systemic route, tick-borne pathogens are transmitted during the blood meals from feeding ticks to a host, establishing systemic infections, and are then transmitted back to any other feeding ticks [37]. The detection of JMTV in both ticks and host blood (cows/buffaloes) suggests the capacity of this virus for systemic infection. The second route is non-systemic transmission via co-feeding. Co-feeding transmission occurs when the pathogen is transferred between infected and naïve vectors that feed in close spatiotemporal proximity on a host that has not yet developed a systemic infection [38, 39]. No data currently exist for JMTV co-feeding transmission.

Our findings showed that only 40.4% of the ticks collected from JMTV-exposed hosts were infected (Table 3), with *R. microplus* demonstrating a marginally significant tendency for higher infection rates (*p*-value = 0.06). While we did not directly confirm cattle infection in our study, recent evidence supports both systemic and potentially non-systemic transmission pathways for JMTV. Viral RNA has been detected in skin biopsies from patients with acute infection following tick bites in China [16], suggesting the theoretical possibility of non-systemic transmission during co-feeding. Additionally, viral RNA has been detected in blood samples from cattle harboring JMTV-positive ticks [28], indicating systemic infection with viremia in peripheral blood. Given this evidence, we propose that *R. microplus*, as a one-host tick species that completes all life stages on a single host over 3–4 weeks, may have enhanced viral acquisition opportunities through prolonged exposure during potential viremic periods, in contrast to two-host *A. variegatum* adults that attach after feeding on different (typically smaller) hosts during immature stages. Additionally, *R. microplus* may exhibit greater intrinsic susceptibility to JMTV infection due to its biological characteristics. These results underscore the need for controlled vector competence experiments to elucidate the relative contributions of different transmission mechanisms in JMTV maintenance.

However, conducting such controlled vector competence experiments requires viral strains, posing certain challenges in the case of JMTV. Since its discovery, numerous attempts to isolate JMTV in various arthropod and mammalian cell lines (Vero, Vero E6, BHK-21, DH82, C6/36 and BME26) have proved largely unsuccessful [3, 9, 16, 40, 41]. To date, stable JMTV propagation has only been achieved in tick-derived cell lines, including BME/CTVM23 (originating from *R. microplus*) [16], ISE6 and IRE/CTVM19 (derived from *Ixodes scapularis* and *I.* *ricinus*, respectively) [42]. In our study, we successfully demonstrated JMTV replication in the HAE/CTVM8 cell line established from *H. anatolicum* ticks.

These virological findings are consistent with field observations of JMTV detection in multiple tick species, including *Hyalomma* spp. [43], suggesting that the virus exhibits broad adaptability across ixodid species. Similar patterns have been observed for related Jingmen group viruses. For example, Alongshan virus (ALSV), which is naturally found mainly in *I. ricinus* and *Ixodes persulcatus* ticks [1, 14, 44–46], has been successfully cultured in both *I. ricinus* (IRE/CTVM19) and *H. anatolicum* (HAE/CTVM8) cell lines [15, 47], despite no natural *Hyalomma* associations being reported. Likewise, Yanggou tick virus (YGTV), primarily detected in *Dermacentor* spp. and *I.* *persulcatus* ticks, has shown replicative capacity in *I.* *ricinus* and *H.* *anatolicum* cell lines [15, 47]. Notably, ALSV and YGTV replication in these cell lines occurred without apparent cytopathic effects. Collectively, these findings demonstrate that tick-associated Jingmen group viruses (JMTV, ALSV and YGTV) exhibit considerable plasticity in their ability to replicate across different ixodid cell lines, regardless of their natural vector associations. This broad cellular tropism is likely to facilitate their geographic expansion and emergence in new ecological niches.

All the Guinean JMTV sequences (both newly obtained and previously reported ones) form a monophyletic cluster, with intragroup phylogenetic distances ranging from 0.0077 to 0.0102 across all four segments. Among other African isolates, the Cameroonian sample is grouped within this West African cluster, while East African strains (from Kenya and Uganda) show marked divergence instead of clustering with Asian isolates (China, Japan and Laos). This phylogenetic pattern correlates with the livestock trade networks, as discussed above. These findings underscore the critical importance of quarantine measures for livestock movement in tropical regions.

Our study revealed low genetic diversity of JMTV circulating in Guinea. This low diversity is particularly striking when compared to Chinese JMTV strains. The analyzed set of Chinese isolates exhibits significantly higher diversity, suggesting that the viral population in China is large enough to avoid genetic drift and has been circulating long enough to accumulate numerous variable sites and unique genetic variants. The low genetic diversity in Guinea is likely to reflect both recent introduction and population bottleneck effects. Additionally, the limited diversity could be attributed to stochastic processes in a small population or the presence of purifying selection. However, our Tajima’s* D* calculations showed no significant deviations from neutrality, preventing us from conclusively confirming demographic causes for the observed low diversity. This result should be interpreted with caution as there may exist potential sampling bias. For whole-genome sequencing, researchers might have selected samples from different tick species, different cattle, different herds or different regions, avoiding geographically close specimens. Nevertheless, our analysis included 82 Guinean JMTV sequences of segment 3, featuring not only haplotypes represented by a single isolate but also haplotypes with 10, 11 or 22 isolates. This gives us confidence that our results are not strongly affected by sampling bias.

On the other hand, our haplotype analysis provided further evidence that the low genetic diversity of JMTV in Guinea is due to the population’s recent origin and a bottleneck event. The haplotype network of Guinean isolates displays a clear star-like topology, typical of recently emerged populations.

In addition to revealing low genetic diversity, our haplotype analysis uncovered important biological features of JMTV circulation that may facilitate diversity generation. A key finding was the detection of ticks carrying different viral genotypes simultaneously feeding on the same host animal. Such co-feeding behavior effectively expands the founding population's genetic diversity, partially mitigating the bottleneck effect typically associated with viral introductions.

The co-occurrence of genetically distinct viral variants in co-feeding ticks, and in particular within individual tick specimens, creates suitable conditions for reassortment events. While we detected no natural reassortants among the samples in this study, we confirmed reassortment in segment 3 for a Guinean isolate reported by other authors. Segment 3 encodes non-structural protein 2 (NSP2), which is homologous to the orthoflavivirus NS3-NS2B complex, exhibiting protease and helicase activities crucial for viral replication. Reassortment in JMTV was first described in the original paper reporting its discovery [3], where evidence of segment 1 replacement in three tick samples and segment 3 replacement in one tick sample was found. These findings corroborate our results and further demonstrate the co-circulation of distinct genetic variants in endemic areas. Importantly, they highlight the role of tick co-feeding behavior in maintaining JMTV genetic diversity.

The putative reassortment candidate identified in our study, isolate KITV2022/4, exhibits the following substitution profile relative to sequences KITV2022/3-2 and KITV2022/3-3: segment 1, 10 substitutions; segment 2, three substitutions; segment 3, zero substitutions; and segment four to six substitutions. We interpret this pattern as evidence of a reassortment event wherein KITV2022/4 acquired segment 3 from viruses identical to KITV2022/3-2 and KITV2022/3-3. However, the relatively low substitution counts in segments 1, 2 and 4 could alternatively suggest these nucleotide changes resulted from random mutational processes rather than reassortment. Several critical observations must be considered when evaluating this possibility.

First, our analysis revealed that segment 3 demonstrated the highest variability among JMTV sequences in this study. This finding creates a paradox when attempting to explain the substitution pattern between KITV2022/4 and the KITV2022/3-2/KITV2022/3-3 group through random mutagenesis—such a process would presumably affect all four genomic segments uniformly, yet we observed complete conservation in segment 3. This inconsistency renders the random mutation scenario biologically implausible. Second, we have established the biological plausibility of reassortment through two key findings: the co-circulation of distinct JMTV genetic variants in ticks collected from a single host animal and, more significantly, the detection of two genetically distinct JMTV variants within individual ticks. These observations confirm the necessary ecological conditions for reassortment to occur naturally. Third, interpretation of reassortment among Guinean sequences must account for the limited genetic diversity demonstrated in our phylogenetic analysis. The viral population diversity is so constrained that segment exchanges between different viral lineages may escape detection, since the minimal substitution counts could mimic random mutation patterns, thereby masking true reassortment events.

Considering these lines of evidence collectively, we infer that the phylogenetic pattern observed across all four segments in isolate KITV2022/4 is most likely to result from reassortment with acquisition of segment 3 from the KITV2022/3-3/KITV2022/3-2 genetic variant. The absence of substitutions in segment 3, contrasted with the substitution patterns in other segments, provides particularly compelling support for this conclusion.

## Conclusions

We have identified at least three JMTV natural foci in Guinea. The primary vectors in these foci are ticks of the *Boophilus* subgenus (notably *R.* *microplus*) and *A.* *variegatum*. As dominant livestock parasites in West Africa with biological traits favoring dispersal, these tick species can readily spread via livestock trade networks, underscoring the critical need for quarantine measures during both domestic and transboundary cattle movements. This study demonstrates low genetic diversity in Guinean JMTV strains, indicating recent emergence with population bottleneck effects. Despite this constrained diversity, the biological characteristics of the virus, and in particular its capacity for reassortment, may facilitate future genetic diversification.

## Supplementary Information

Additional file 1. Table S1. Primers used for JMTV amplification and sequencing.** Table S2. **Within-group genetic distancesfor each segment of JMTV.

Additional file 2. Table S1. List of sequences of JMTV used for phylogenetic analysis.

Additional file 3. Figure S1. Sanger sequencing chromatograms of JMTV genomic fragments showing heterogeneous nucleotide positions. Numbers indicate nucleotide positions relative to reference sequences: MW341210, MW341211, MW341212and MW341213.

## Data Availability

The data supporting the findings of this study are publicly available from GenBank with the identifiers PP779169–PP779192, and PV461569–PV461589. JMTV primer sequences are provided in Additional file 1: Table S1. The vector layers of the maps in Figs. 1 and 3a were downloaded from Natural Earth public domain (https://www.naturalearthdata.com/downloads/10m-physical-vectors/).

## Abbreviations

ALSV: Alongshan virus
JMTV: Jingmen tick virus
TAKV: Takachi virus
YGTV: Yanggou tick virus
SD: standard deviation
95% CI: confidence intervals
