# Supplementary material for: The low genetic diversity of the Jingmen tick virus in Guinea sheds light on the recent introduction of the virus to West Africa
Source: Parasit Vectors. 2025 Nov 4;18:446. doi: 10.1186/s13071-025-07089-z (PMC12584544; doi:10.1186/s13071-025-07089-z)
Supplement: Supplementary file 1 — Additional file 1. Table S1. Primers used for JMTV amplification and sequencing. Table S2. Within-group genetic distancesfor each segment of JMTV. [file 13071_2025_7089_MOESM1_ESM.docx]

# Additional file 1:

Table S1. Primers used for JMTV amplification and sequencing

| Primer | Sequence | Start position* | End position* | Amplification program |
| --- | --- | --- | --- | --- |
| Segment 1 | | | | |
| Kind-NS5-F-20 | ACTGCAAGTGCAAAGGTTGAGGA | 8 | 30 | 95ᵒ-5’  95ᵒ-10’’  59ᵒ-10’’ х 44  72ᵒ-40’’  72ᵒ-2’ |
| Kind-NS5-R-820 | CCTTCTTAGTCTTCACTTGCCAC | 811 | 833 |  |
| Kind-NS5-F-730 | GTAGACGCATGGATGATGCAG | 716 | 736 |  |
| Kind-NS5-R-1490 | CCTAAGTCTCCGTAGTTCCTG | 1477 | 1497 |  |
| Kind-NS5-F-1380 | ATCCTCGCCGATCTCTGGCT | 1367 | 1386 |  |
| Kind-NS5-R-2200 | AGAGGACCTTGTCATCCCCCGA | 2174 | 2195 |  |
| Kind-NS5-F-2080 | GACAACATACGGGATGGCCGT | 2071 | 2904 |  |
| Kind-R-2900 | ACGGTGGCGCTAACACCGCA | 2904 | 2923 |  |
| Segment 2 | | | | |
| Kind-Gly-F-130 | GCTAAGACAAGACAAGAATGGCA | 131 | 153 |  |
| Kind-Gly-R-830 | ACGTACACTCTGTCAGTGACGGT | 830 | 852 |  |
| Kind-Gly-F-700 | CTACGGATGGGACTGTGACGA | 697 | 717 |  |
| Kind-Gly-R-1420 | TGGTCTGATACACATCCTTGGCA | 1423 | 1445 |  |
| Kind-Gly-F-1310 | GAGGTCACTCGGTACCACGAG | 1313 | 1333 |  |
| Kind-Gly-R-2070 | TGCCAAAGGCCTCGGATGGGT | 2065 | 2085 |  |
| Kind-Gly-F-1940 | TGGCGTTGCCCTCTACCACA | 1942 | 1961 |  |
| Kind-Gly-R-2740 | ACTTGCCAGCCATATGGAGGA | 2699 | 2719 |  |
| JMTV_test_F1 | GAAGTACTCCATCTACACCAGA | 781 | 802 | 95ᵒ-5’  95ᵒ-10’’  50ᵒ-10’’ х 40  72ᵒ-40’’  72ᵒ-2’ |
| JMTV_test_R1 | CACGGCTCCCTACTCATG | 1159 | 1176 |  |
| Segment 3 | | | | |
| Kind-NS3-F-40 | GCGCTAGACTCACTTTACAGACT | 29 | 51 | 95ᵒ-5’  95ᵒ-10’’  59ᵒ-10’’ х 44  72ᵒ-40’’  72ᵒ-2’ |
| Kind-NS3-R-740 | TCAATCCAAACCGCTCTGTCC | 718 | 738 |  |
| Kind-NS3-F-640 | AGCACATCGGACCGTGGGCT | 636 | 655 |  |
| Kind-NS3-R-1330 | GACYACTCTGGCCAGAGCATG | 1310 | 1330 |  |
| Kind-NS3-F-1210 | AGGCAACTCATGACCTGGTCCA | 1181 | 1202 |  |
| Kind-NS3-R-2070 | CTAAGGCACATGTTGGCCTCCA | 2055 | 2076 |  |
| Kind-NS3-F-1950 | CTCAGACCTGCCAGAGGAGA | 1935 | 1954 |  |
| Kind-NS3-R-2640 | CGCTTGCCGCAACCTAGTCA | 2624 | 2643 |  |
| Segment 4 | | | | |
| Kind-Seg4-F-30 | CGTCGCAGACTCAATCGAACGA | 33 | 54 | 95-5’  95-10’’  59-10’’ х 44  72-40’’  72ᵒ-2’ |
| Kind-Seg4-R-790 | GATTGTGTAGTGGCCCTTCGTCA | 792 | 814 |  |
| Kind-Seg4-F-660 | GATCACATGGTAGCAATACACCCT | 665 | 688 |  |
| Kind-Seg4-R-1420 | AGGACTTGGTTGGTAGTAGTGGA | 1415 | 1437 |  |
| Kind-Seg4-F-1260 | ACTACTACGTAGCCATCATGCTG | 1256 | 1278 |  |
| Kind-Seg4-R-2080 | AGGTGGTAAGTGGGTACCATCA | 2084 | 2105 |  |
| Kind-Seg4-F-1960 | GAAGATCACACTCGCTGTCAGCA | 1962 | 1984 |  |
| Kind-Seg4-R-2700 | GAACGAGATCAGGGCACTCACT | 2696 | 2717 |  |

* позиция первого нуклеотида праймера относительно референса:

MW341210 – for Segment 1;

MW341211 – for Segment 2;

MW341212 – for Segment 3;

MW341213 – for Segment 4.

Table S2. Within group genetic distances (p-distance) for each segment of JMTV

| Statistic | Segment 1 | Segment 2 | Segment 3 | Segment 4 |
| --- | --- | --- | --- | --- |
| Guinean sequences (n = 18) | | | | |
| Median | 0.0108 | 0.0086 | 0.0127 | 0.0102 |
| Mean | 0.0087 | 0.0077 | 0.0099 | 0.0102 |
| SD | 0.0054 | 0.0054 | 0.0066 | 0.0048 |
| Chinese sequences (n = 36) | | | | |
| Median | 0.0535 | 0.0499 | 0.0594 | 0.0626 |
| Mean | 0.0384 | 0.0393 | 0.0399 | 0.0401 |
| SD | 0.0297 | 0.0317 | 0.0299 | 0.0347 |
