# Supplementary figures and images for: The low genetic diversity of the Jingmen tick virus in Guinea sheds light on the recent introduction of the virus to West Africa
Source: Parasit Vectors. 2025 Nov 4;18:446. doi: 10.1186/s13071-025-07089-z (PMC12584544; doi:10.1186/s13071-025-07089-z)

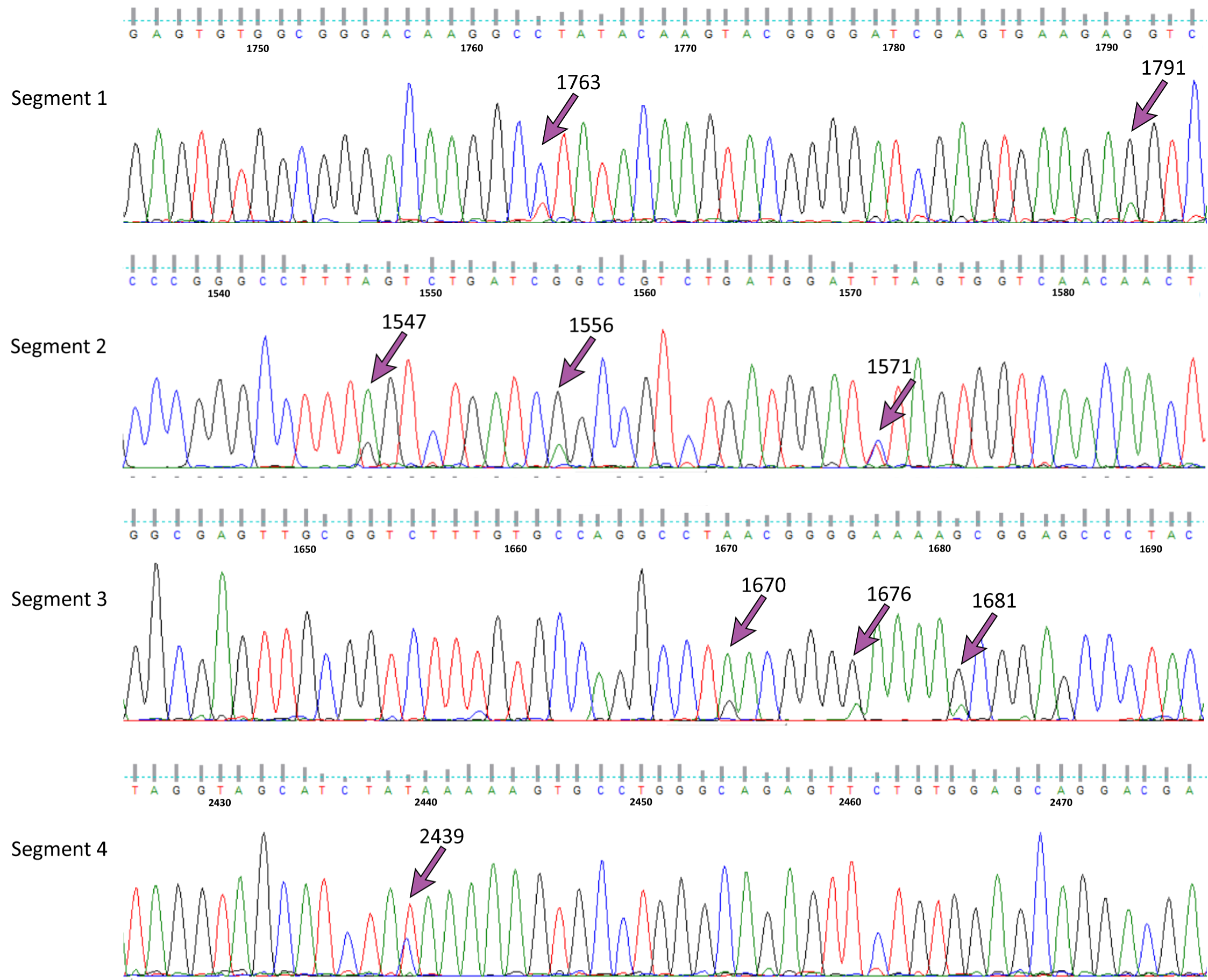

Supplement: Supplementary file 3 — Additional file 3. Figure S1. Sanger sequencing chromatograms of JMTV genomic fragments showing heterogeneous nucleotide positions. Numbers indicate nucleotide positions relative to reference sequences: MW341210, MW341211, MW341212and MW341213. [file 13071_2025_7089_MOESM3_ESM.tif]
